# Performance of Sustainable Green Concrete Incorporating Quarry Dust and Ferronickel Slag as Fine Aggregate

**DOI:** 10.3390/ma17102326

**Published:** 2024-05-14

**Authors:** Md Nuruzzaman, Jaydon Almeida, Md Tanvir Ehsan Amin, Prabir Kumar Sarker

**Affiliations:** School of Civil and Mechanical Engineering, Curtin University, GPO Box U1987, Perth, WA 6845, Australia; jaydon.almeida@graduate.curtin.edu.au (J.A.); m.amin6@postgrad.curtin.edu.au (M.T.E.A.)

**Keywords:** quarry dust, ferronickel slag, mechanical properties, microstructure

## Abstract

This paper presents a study on the combined use of two by-products, namely quarry dust (QD) and ferronickel slag (FNS), as a full substitute for natural sand to improve the greenness of concrete production. Quarry dust was used in increments of 25% to a maximum of 75% substitution, where nickel slag was used as the remaining proportion of fine aggregate. All the combinations of quarry dust and nickel slag were found to be compliant with AS 2758.1 and they showed notably better grading than 100% sand. In this research, standard concrete tests, such as the slump test for fresh concrete, and compression, tensile and shrinkage tests for hardened concrete, were conducted. Scanning electron microscopy and X-ray diffraction analysis were also conducted for microstructural investigation. The results concluded that the combinations of quarry dust and nickel slag in concrete as a whole substitution of sand provide similar results for these properties. Specifically, 25% quarry dust with 75% nickel slag proved to be the most promising alternative to sand, with compressive and splitting tensile strengths of 62 and 4.29 MPa, respectively, which were 16% and 20% higher than those of the control mix. Also, lower drying shrinkage was observed for this combination compared to the control mix. The higher strength is attributed to the rough texture and angular shape of both quarry dust and nickel slag providing a better mechanical interlocking. The validity of this result has also been confirmed through image analysis of micrographs from various specimens. In microstructural investigations, specimens with QD and FNS exhibited fewer voids and a more compact surface compared to the control specimen. This shows the potential for further research into the use of quarry dust and nickel slag in the production of green concrete.

## 1. Introduction

Concrete consists of a combination of a binder, water and aggregate. Aggregate serves as the filler and is divided into fine aggregate (FA) and coarse aggregate (CA). According to Australian standard AS 2758.1 [1], FA is defined as an aggregate with the majority of particles passing a 4.75 mm AS sieve, which is sourced either from naturally occurring sand deposits or manufactured by the crushing and processing of rocks. Fine aggregate constitutes 35% to 45% of concrete’s volume [2]. Natural sand is the most used fine aggregate and is best obtained from riverbeds as it requires less processing and possesses better quality in terms of particle size distribution and texture. Excessive sand dredging may cause physical disturbance to the riverbed and change in the flow, and affect the aquatic life and the surrounding communities [3]. For example, a case study on sand dredging at the western Baltic Sea reported a 2-to-4-year recolonization period for respective fauna and noted that biomass after a 2-year period was still reduced in comparison to the pre-dredged state. Results derived from a study by Boyd et al. [4] concluded that the effects of high and low dredging intensity are evident after 6 years of dredging. 

Moreover, due to rapid industrialisation, the demand of concrete is increasing rapidly as well [5]. With over 4.4 billion tonnes of concrete produced per year globally [6], natural sand is quickly becoming limited and expensive. According to The Japan Times, in 1990, Japan banned marine sand mining and developed alternate methods for fine aggregate production by crushing quarry waste [7]. Given that the associated economic and environmental impact of sand dredging are becoming increasingly significant, and the subsequent effect has resulted in a national ban, it is critical that other sources of fine aggregate are investigated for the purpose of providing a partial or whole replacement of sand as fine aggregate in concrete production. Indeed, various alternatives to natural fine aggregates have been suggested, including the utilization of plastic aggregates or graphene oxide [8,9]. On a global scale, research into using by-products as fine aggregate replacement can create a basis for long-term cost and environmental benefits in the construction industry.

Research into by-products as a partial or whole substitute of sand has been studied substantially. Approximately 20 to 25 percent of the overall production in every crusher unit is designated as quarry waste [10]. This waste, known as quarry dust (QD), stems from the stone crushing industry and is abundantly generated, reaching approximately 200 million tonnes annually [11]. Wellala [12] presents the properties of concrete using quarry dust as a partial substitution of sand. The authors found a significant drop in slump with the increase in percentage replacement of sand by crushed granite aggregate. The authors also reported a slightly higher compressive strength for the concrete incorporating quarry dust than the control. The 28-day compressive strength was reported as maximum when using 60% sand was substituted by quarry dust. Conversely to compressive strength, tensile strength showed a declining trend with the sand replacement by crushed fine aggregate. In a different study, Muhit et al. [13] found that replacing 35% sand by quarry dust in mortar improved compressive strength by 20% and tensile strength by 13.5% in comparison to control mortar. Again, Nuruzzaman et al. [14] used this supplementary fine aggregate in concrete and recorded that 30% sand replacement by QD increases the compressive strength by 15% and tensile strength by 10%. This study also showed that the compressive strength of concrete increased for up to 30% sand replacement by QD and declined for 40% sand replacement. On the other hand, Nickel slag is a by-product of Nickel alloy production. About 10 to 12 tons of slag is produced as a by-product in the production of one ton of nickel. Saha and Sarker [15] tested concrete using ferronickel slag (FNS) as a sand substitution. The 12% increase in slump in comparison with the control batch is attributed to the decrease in surface area of FNS. The authors also noted that there was an increase in compressive strength when nickel slag substitution was increased from 0% to 50%. Compressive strength was maximum at 50% substitution, with a value of 66.28 MPa, and 8% greater than concrete using 100% natural sand. This increase was observed in another study by Saha et al. [16] that used non-destructive methods to predict the compressive strength of concrete of the same mix proportions to be 73 MPa and 84 MPa for 0% and 50% nickel slag replacement, respectively. FNS had also been used in self-compacting concrete [17,18]. Research findings showed promising results as 40% sand replacement by FNS increased the compressive strength by around 30% and the splitting tensile strength by around 15% [19]. Leaching of heavy metal from FNS was found to be well below the permitted limits [20]. Results from Shoya et al. [21] revealed that shrinkage reduced for 50% and 100% sand substitution by nickel slag in comparison to the control mix.

Thus, the above studies show that the combined use of quarry dust and nickel slag as whole fine aggregate substitution in concrete has a strong base for success. Currently, no research has investigated the properties of concrete using a combination of quarry dust and nickel slag as fine aggregate. Therefore, the fresh and hardened properties of concrete using different proportions of these two by-products were studied to investigate their suitability as a replacement of natural sand to produce greener concrete. 

## 2. Materials and Methods

### 2.1. Materials

#### 2.1.1. Binder

In this research, ordinary Portland cement (OPC), complying with AS 3972 [22] and ASTM C150 [23], was used as the binder. The chemical composition of OPC is generally enriched with CaO, SiO_2_ and Al2O_3_ [17].

#### 2.1.2. Fine and Coarse Aggregates

**Figure 1:**
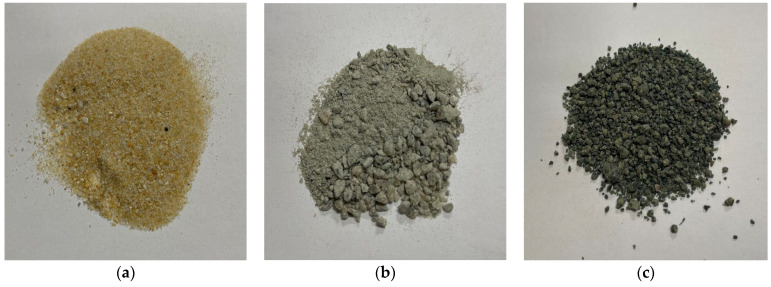
Physical appearance of (**a**) sand, (**b**) QD and (**c**) FNS.

**Figure 2:**
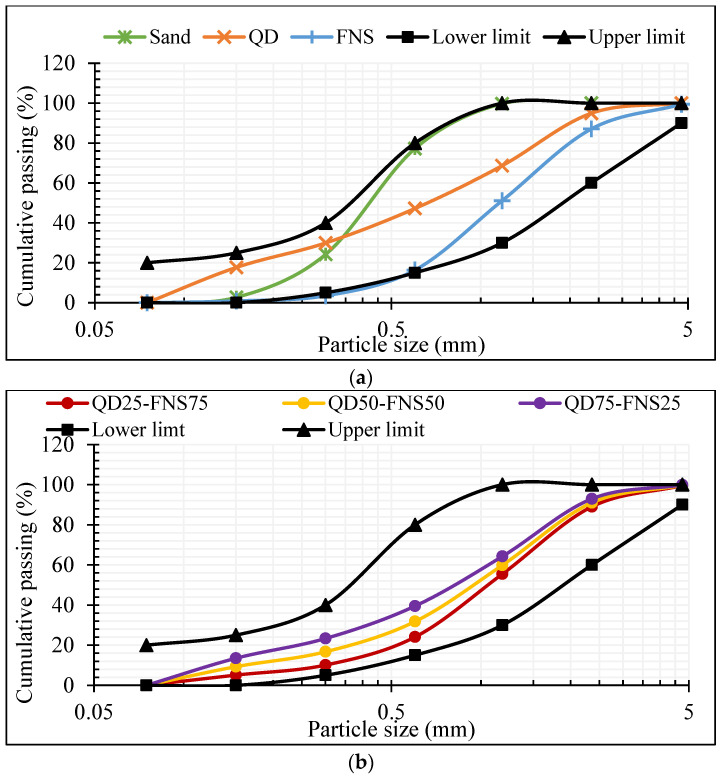
Particle size distribution of fine aggregates, (**a**) Sand, QD and FNS, (**b**) QD25-FNS75, QD50-FNS50 and QD75-FNS25.

In this research, natural sand, ferronickel slag and quarry dust were used as fine aggregate and crushed granite with a maximum size of 20 mm was used as a coarse aggregate. The physical appearance of fine aggregates is given in Figure 1. From the figure, it is apparent that both the QD and FNS had coarser particles than the natural sand. Moreover, QD and FNS are angular in shape in comparison to round-shaped natural sand. The particle size distribution (PSD) of the fine aggregates was determined in accordance with AS 1141.11.1 [24]. Given that the upper limits for fine aggregate are different for natural fine aggregate and manufactured aggregate as per AS2758.1 [1], the upper limits therefore are different for each combination of quarry dust and nickel slag. For clarity of compliance, particle size distribution curves for all the fine aggregates and their combinations are shown in Figure 2. In Figure 2b, Mix QD25-FNS75 denotes a blend comprising 25% quarry dust and 75% ferronickel slag. Similarly, this pattern applies to all other mixes.

Based on the data used to create Figure 2a, it was found that sand has a much narrower distribution in comparison to quarry dust and nickel slag, with 53% of mass retained on the 300 μm sieve. Maximum retention of quarry dust was noted on the 1.18 mm sieve, with 26.3% of mass retained. Similarly, nickel slag had a maximum 35.9% of mass retained on the 1.18 mm sieve; however, it also had a retained percentage of 34.7% on the 600 μm sieve. These data show that quarry dust and nickel slag have a more even distribution of grading compared to sand. Analysing the upper and lower limits as per AS 2758.1 [1], natural sand and quarry dust are within limits and therefore compliant. The distribution curve for nickel slag falls under the lower limit for sieve sizes 1.18 mm down to 300 μm, and therefore indicates that FNS should not be used as a whole substitute for sand, limited to a partial replacement. Displayed in Figure 2b, the combinations of quarry dust and nickel slag provide more well-graded distributions in comparison to natural sand, quarry dust and nickel slag as a singular fine aggregate. All distributions were compliant, and therefore, these combination percentages were studied in this research. 

The fineness modulus (FM) of an aggregate is a measure of the coarseness of the particles. A higher FM means a generally coarser aggregate. Shown in Table 1, both quarry dust and nickel slag had an FM greater than sand. Interpreting these data in conjunction with the graph shown in Figure 2 indicates that the combination of quarry dust and nickel slag as fine aggregate provides a more well-graded particle distribution than natural sand.

The water absorption and specific gravity of the aggregates are shown in Table 2. The standard procedure for determining the particle density and water absorption, detailed in AS 1141.5 [25] for fine aggregate and AS 1141.6.2 [26] for coarse aggregate, was followed. Nickel slag had a density of 2815 kg/m^3^, which was the highest among all. Water absorption values were consistent across all aggregates, with quarry dust having the highest absorption of 3.00%. The dry particle density values for both fine aggregate were within the range of 2100 kg/m^3^ to 3200 kg/m^3^, and therefore classified as normal-weight aggregate in accordance with AS 2758.1 [1].

### 2.2. Concrete Mix Details

The concrete mix details are given in Table 3. The mixtures were designated by the percentages of QD and FNS. For example, Mix QD25-FNS75 represented a combination of 25% quarry dust with 75% ferronickel slag. 

### 2.3. Concrete Mixing and Casting 

The concrete batches were mixed using a 70-litre mixer. Prior to mixing, the drum was cleaned thoroughly and dried to ensure no contamination of the sample from previous uses of the mixer. The aggregate was mixed in the drum for three minutes to create aggregate consistency before adding the cement and water. After the addition of water and cement, the mixer ran for another two minutes before the slump test commenced. After casting, all specimens were compacted using a vibrating table with controlled time and amplitude of vibration to avoid any segregation of aggregate. The goal was to remove air bubbles while maintaining the aggregate in suspension. The specimens were removed from the moulds 24 h after casting and subsequently cured in lime water at a temperature of 23 °C until testing. Three cylinders measuring 100 mm × 200 mm were employed to ascertain the average compressive strength, while two cylinders sized 150 mm × 300 mm were utilized for assessing the splitting tensile strength. On the other hand, for the drying shrinkage test, three 75 mm × 75 mm × 285 mm rectangular prism-shaped specimens were used. 

### 2.4. Test Methods

#### 2.4.1. Fresh Concrete Testing

The slump test was conducted in accordance with AS 1012.3.1 [27], immediately after mixing the concrete. Each mix underwent the test twice. This test was recorded once per batch due to the volume of concrete required for testing and the fact that it cannot be reused. Slumps that collapsed laterally were not considered for the results and the test was repeated.

#### 2.4.2. Mechanical Properties

##### Uniaxial Compressive Strength

In accordance with AS 1012.9 [28], the samples were cured in lime-saturated water for periods of 7 and 28 days before testing. In order to ensure that the testing machine applied an evenly distributed load, all samples were sulphur-capped. This was allowed to cool for 4 h prior to testing to mitigate failure due to cracking of the sulphur cap causing uneven load. Load was applied constantly at a rate of 0.33 MPa/second until failure of the specimen. Three specimens were tested from each mixture at each testing age, and the average values are reported. 

##### Splitting Tensile Strength

The splitting tensile strength test was conducted as per standard AS 1012.10 [29]. The cured cylinders of 150 mm diameter and 300 mm height were placed horizontally in the MCC8 testing machine and load was applied perpendicular to the cylindrical face at a rate of 0.067 MPa/second. Two specimens were tested from each mixture at each testing age, and the average values are reported. 

#### 2.4.3. Drying Shrinkage

The samples were cured in lime-saturated water for 7 days prior to testing. Once the initial 7 days measurement was taken, all samples were then placed in a temperature and humidity-controlled chamber compliant with AS 1012.8.4 [30]. The length measurement of the samples was conducted over a period of 49 days.

#### 2.4.4. Microstructural Investigation

The microstructures were examined using scanning electron microscopy (SEM) and energy-dispersive X-ray spectroscopy (EDS). This procedure was carried out using a MIRA VP-FESEM. For this test, backscattered electron mode and secondary electron mode were both employed. At least five SEM images and their associated EDS spectra were gathered for each sample and images were obtained at 20 kV. The aforementioned techniques investigated the elemental makeup of the concrete’s surface topography. The X-ray diffraction (XRD) examination was employed to determine the crystalline structure. The samples were examined using a copper K alpha radiation source (40 kV and 40 mA) and a LynxEye XE-T detector on the powder diffractometer D8 Advance (Bruker AXS, Germany). The spectra were gathered between 2θ values of 5 and 120°.

## 3. Results and Discussion 

### 3.1. Fresh Properties

**Figure 3:**
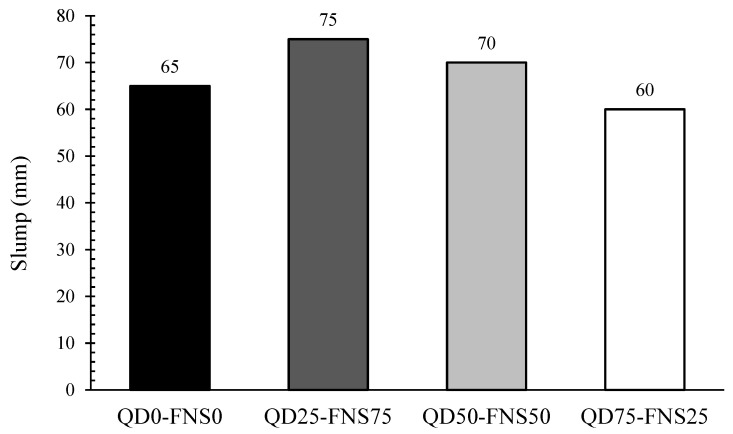
Slump values of different mixes.

The workability of fresh concrete was determined using a slump test. The results for each batch are plotted in Figure 3. The three combinations of quarry dust and nickel slag as a full substitute are compared with the control batch, consisting of 100% natural sand. Slump values varied in a narrow range from 60 mm to 75 mm, where the control had a slump of 65 mm.

As the particle size increases, the total surface area of the aggregate that water is required to saturate decreases. Therefore, as the particle size increases, the viscosity of concrete decreases and workability increases [31]. This is evident in Figure 3, where QD25-FNS75 has a slump 15.4% greater than the control. As discussed in the previous section, nickel slag had a fineness modulus of 3.42 whereas sand had a FM of 1.96. Quarry dust had an FM of 2.42. This same trend was observed in the slump of QD50-FNS50, which was approximately 7% greater than the control batch and 7% less than QD25-FNS75. This was expected as the proportion of quarry dust increased to 50%. The trend continued with the mixture QD75-FNS25; however, it showed a slump less than that of QD0-FNS0. This drop can be explained by the shape of the particles. This is attributed to the angular shapes of QD and FNS in comparison to the round shape of natural sand. The angularity and rough texture of quarry dust and nickel slag result increased the resistance to flow. This resistance resulted in less workability of mixture QD75-FNS25. 

### 3.2. Mechanical Properties

#### 3.2.1. Compressive Strength

**Figure 4:**
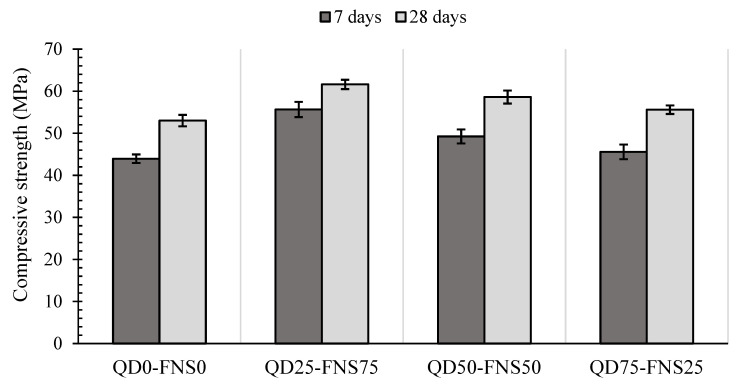
Compressive strength of different mixes.

The 7- and 28-day compressive strengths are presented in Figure 4. The QD25-FNS75 batch had a notably higher 7-day compressive strength over the control batch, with a 27% increase. The QD50-FNS50 batch, similarly, had an increase in compressive strength over QD0-FNS0, with a 12% increase, recording a 7-day compressive strength of 49.25 MPa. The QD25-FNS75 compressive strength at 7 days was similar to QD0-FNS0, with an increase of 1.63 MPa.

The 28-day compressive strength of mixture QD25-FNS75 was higher than that of QD0-FNS0, with an increase of 16%. Similar to the trend for the 7-days strength, QD50-FNS50 had a 28-day compressive strength 11% greater than QD0-FNS0than that of QD0-FNS0 and slightly lower than that of QD25-FNS75. Mix QD75-FNS25 had a 28-day compressive strength 5% higher than QD0-FNS0, similar to the trend of 7-day strength.

The graph displayed in Figure 4 shows a strength gain for QD0-FNS0, QD50-FNS50 and QD75-FNS25 from 7 days to 28 days of 21%, 19% and 22%, respectively, whereas for QD25-FNS75, the strength gain was lower, 11%, with a strength increase of 5.95 MPa. Coarser fine aggregate may experience less early age shrinkage compared to finer aggregates due to reduced water demand and internal restraint. The reduced shrinkage for QD25-FNS75 can be confirmed by the findings presented later in Section 3.3. This reduced shrinkage can contribute to better preservation of internal cohesion and strength at early ages, resulting in higher compressive strength at 7 days than the strength at 28 days. The 24 samples analysed for both 7- and 28-day compressive strength experienced a standard deviation range of 1.01 to 1.73. These standard deviations are also shown in Figure 4. The low range and low values of deviation indicate that the experimental data obtained for compressive strengths of all batches are reliable with low variability. The maximum variation for individual specimens was 4.09 MPa for the 7-day compressive strength of QD25-FNS75. The strength increase in all combinations of quarry dust and nickel slag in comparison to the control batch can be explained using the PSD graphs plotted in Figure 2. 

Whole substitution of sand with quarry dust and nickel slag provided a well-graded distribution of aggregate, with a fineness modulus of 3.17, 2.92 and 2.67 for QD25-FNS75, QD50-FNS50 and QD75-FNS25, respectively. As displayed in the aforementioned figures, sand has a limited and narrow distribution of particles, with no particles larger than 2.36 mm. Quarry dust and nickel slag, however, as a combination have a much more even distribution. This even distribution results in a denser packing of particles with less voids. This is aligned with Hewlett [32], who concluded that performance of concrete is significantly impacted if the aggregate used is too fine, with little coarse particles. The study also revealed that grading of fine aggregate impacts the properties of concrete more than coarse aggregate. Given that coarse aggregate grading was consistent for all batches, this conclusion further explains the reason for higher compressive strength of all the batches than the control.

Furthermore, the specific gravity of nickel slag was 2.82, which was about 0.3 more than the specific gravity of sand. Quarry dust had a specific gravity slightly less than sand. This therefore increased the bearing capacity of concrete under compression and can explain why QD25-FNS75 produced a greater 7- and 28-day compressive strength than all other batches.

Additionally, the nature of the quarry dust and nickel aggregate was rough and angular, as shown in Figure 1. In contrast, sand particles had a more spherical shape with a smooth surface. Results of Aitcin [33] showed an increase in flexural, compressive and tensile strength due to the rough surface texture of granite. The rough texture of quarry dust and nickel slag provided a surface with increased friction, and therefore the mechanical interlocking of aggregate was increased, giving the results shown above. This result also correlates with the data reported by Ahn [34], which showed that the bond strength of aggregate with cement increased as the roughness of aggregate increased. 

As observed, QD75-FNS25 recorded the lowest compressive strengths for the quarry dust and nickel slag combinations. Given the ratio of 75% quarry dust, the batch subsequently had the lowest fineness modulus of 2.67. Haque et al. [35] investigated the effect of aggregate size on the compressive strength of concrete and reported an upwards trend in compressive strength as the fineness modulus increased. This explains why QD75-FNS25 had a lower 7- and 28-day compressive strength in comparison to QD25-FNS75 and QD50-FNS50. 

#### 3.2.2. Splitting Tensile Strength

**Figure 5:**
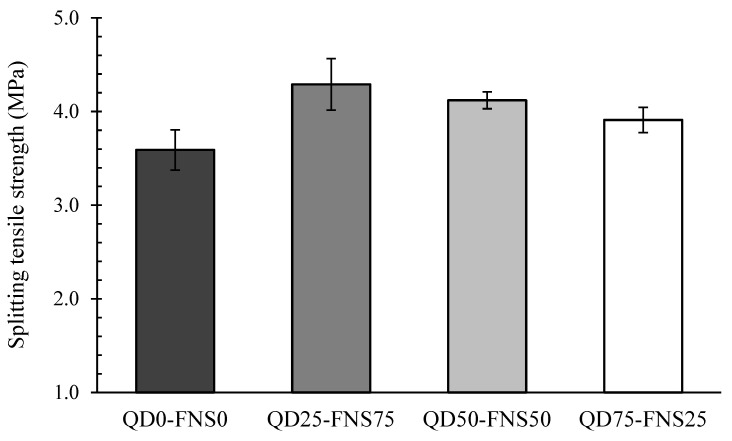
Splitting tensile strengths of different mixtures at 28 days.

**Figure 6:**
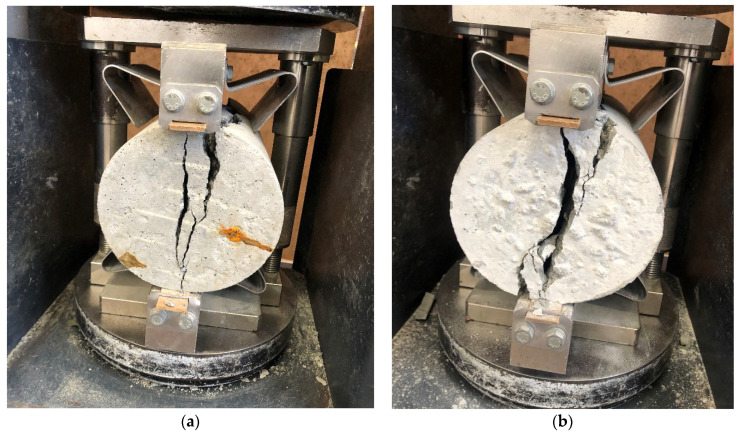
Tensile cracking pattern of (**a**) QD0-FNS0 and (**b**) QD25-FNS75.

The average splitting tensile strength values at 28 days are listed in Table 4 and plotted in Figure 5. The cracking pattern was captured during the experiment and presented in Figure 6. The results for splitting tensile strength varied from 3.59 MPa for QD0-FNS0 to 4.29 MPa for QD25-FNS75. 

The trend noted in Figure 5 was similar to that of the compressive strengths shown in Figure 4. Mix QD25-FNS75 showed the highest tensile strength, similar to compressive strength. The tensile strengths successively decreased as the quantity of quarry dust increased. The control mix had a lower strength than QD75-FNS25, with a difference of 9%.

Mix QD25-FNS75 showed an 11% increase in strength for 28-day compressive strength and a 20% increase in 28-day tensile strength. This increase in strength gain is attributed to the rough texture and angular shape of quarry dust and nickel slag, and therefore an increase in the mechanical interlocking of the aggregate. This interlocking provided a higher resistance to splitting, resulting in a more tortuous cracking pattern. Due to the blocking effect of rough-textured nickel slag, the actual cracking profile was more tortuous for the QD25-FNS75 specimen, whereas the presence of straight cracks and relatively flat fracture surfaces were evident for the QD0-FNS0 specimens, indicating brittle failure traits and a limited capacity for tensile strain. The decrease in tensile strength as the quarry dust content was increased parallels the data form Ukpata and Ephraim [36], which showed a decrease in tensile strength as the quarry dust content was increased. The trend of tensile strength results is similar to that reported by Saha and Sarker [15]. The authors explained the trend using the cracking pattern, which showed an identical cracking pattern to that in Figure 6. Nevertheless, the ultimate formation of cracks varied slightly among the specimens, contingent upon the tensile strain they endured [37]. The conclusion was that the rough texture and angular shape of nickel slag provided a higher resistance to splitting, causing a more tortuous cracking pattern.

#### 3.2.3. Correlation between Tensile and Compressive Strengths

There is a correlation between the splitting tensile strength and compressive strength when natural sand is used as fine aggregate. AS 3600 [38] under Clause 3.1.1.3 has detailed respective equations to predict the splitting tensile strength based on the experimental compressive strength. Since the correlation is definitive, the relationship between tensile strength and compressive strength is expressed as a simple relationship under Clause 3.1.1.3. The equation is as follows:

(1) $${f^{\prime }}_{ct}=0.36\sqrt{{f^{\prime }}_{c}}$$

where *f′_ct_* = characteristic uniaxial predicted tensile strength 

= 0.9 × *f_ct.sp_*

*f′_c_* = characteristic cylinder measured compressive 

strength at 28 days

*f_ct.sp_* = measured splitting tensile strength of concrete

The characteristic strength in Equation (1) was multiplied by 1.4 to obtain the mean value. The calculated variable *f′_ct_* was multiplied by 1/0.9 to convert uniaxial tensile strength to the splitting tensile strengths shown in Table 4. 

The experimental/calculated ratio column displayed values all close to 1. The QD25-FNS75 and QD50-FNS50 batches had the closest ratios of 1.00 and 0.99, respectively, and therefore it can be concluded that the equations listed in AS 3600 [38] can provide a relatively accurate prediction for tensile strength in this study if compression values are known. The opposite can also be said if the tensile strength is known.

### 3.3. Drying Shrinkage

**Figure 7:**
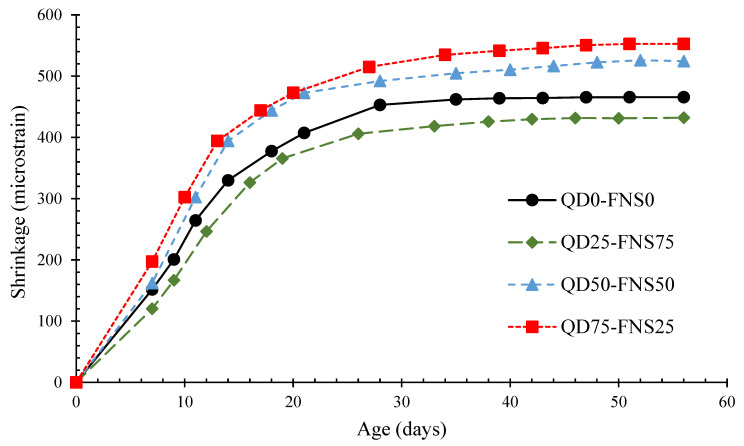
Drying shrinkage of different mixes.

The drying shrinkage values for all batches are shown in Figure 7. For all batches, the shrinkage was distinguished during the early curing days, with a high increase in shrinkage until approximately 28 days. From the age of 28 days to 56 days, the shrinkage plateaued, with minimal increase in shrinkage. 

To ensure reliability, the R^2^ value was measured against a polynomial trend line for each mix, which involved a regression plot. The R^2^ values ranged from 0.9984 to 0.9999, with QD75-FNS25 having the data with the lowest R^2^ value. These values close to 1.00 indicate that the data for shrinkage were statistically reliable. 

The QD75-FNS25 and QD50-FNS50 concrete specimens had a similar shrinkage behaviour, paralleling the microstrain until approximately the 20th day of curing. QD50-FNS50 displayed a similar shrinkage at 7 days of curing, with a variation of 10.52 microstrain. The QD75-FNS25 batch had the highest shrinkage, followed by QD50-FNS50. QD25-FNS75 had the lowest shrinkage, notably below the control batch, QD0-FNS0. Tangchirapat and Jaturapitakkul [39] determined that free water evaporation became dominant for drying shrinkage after exceedance of the replacement ratio critical level. The results in Table 2 show that quarry dust had a significantly higher water absorption than the other aggregates. Therefore, the findings of Tangchirapat and Jaturapitakkul [39] well explain the highest shrinkage for the QD75-FNS25 batch, which had 75% of quarry dust as fine aggregate. This attribution also fit to the batch QD50-FNS50, with the second highest shrinkage. 

QD25-FNS75 had 75% of nickel slag as fine aggregate substitution, and had a water absorption of 0.48%. Natural sand had a water absorption of 0.68%, slightly higher than nickel slag. While the 25% of quarry dust in QD25-FNS75 would certainly impact free water evaporation, the mechanical interlocking of quarry dust and nickel slag due to surface friction and its angular shape explains why QD25-FNS75 had a lower shrinkage than QD0-FNS0. Furthermore, natural sand had a fineness modulus of 1.96, whereas QD25-FNS75 had a fineness modulus of 3.17. This means that there exists a greater surface area of aggregate in the QD0-FNS0 batch, which results in greater exposure to free water in a specimen.

Results from Wellala [12] and Shoya et al. [21] showed similar trends. The increase in shrinkage was explained by Wellala [12] using the water absorption values. The conclusion was that the increase in water absorption for quarry dust resulted in the 60% replacement shrinking more over 56 days than the 40%, 20% and control. The quarry dust used in this research had a water absorption of 3.00%, significantly higher than sand and nickel slag, resulting in the QD75-FNS25 batch having the worst shrinkage performance. The results from Shoya et al. [21] similarly concluded that the use of nickel slag in concrete reduced shrinkage as the percentage substitution increased in comparison to the control batch. It follows that, given natural sand has a water absorption 0.2% greater than nickel slag, the water demand of concrete decreases when the nickel slag content increases.

### 3.4. Microstructural Properties

**Figure 8:**
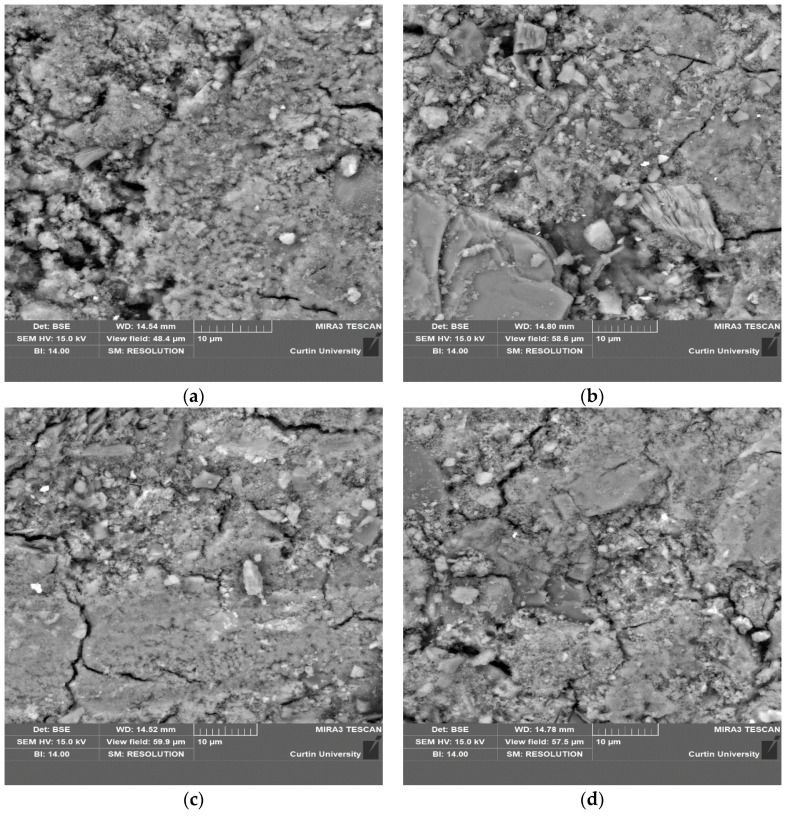
SEM micrographs of (**a**) QD0-FNS0, (**b**) QD25-FNS75, (**c**) QD50-FNS50 and (**d**) QD75-FNS25.

**Figure 9:**
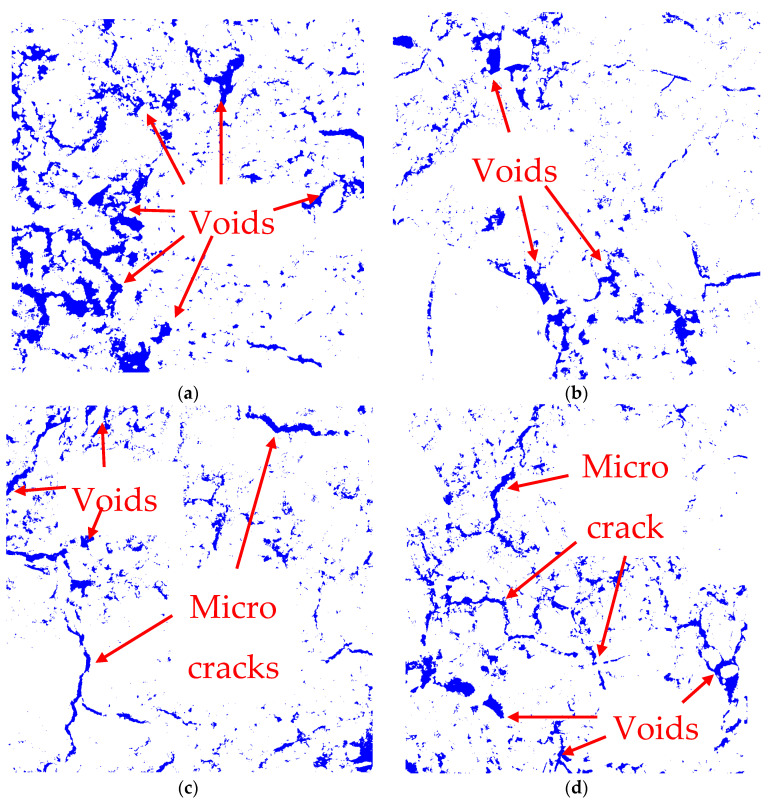
Image analysis of (**a**) QD0-FNS0, (**b**) QD25-FNS75, (**c**) QD50-FNS50 and (**d**) QD75-FNS25.

A microstructural examination using SEM images was conducted with the goal of acquiring a thorough understanding of the compactness and the interfaces of the aggregates. The SEM micrographs of various mixtures are displayed in Figure 8. The same SEM images were subjected to image analysis, as shown in Figure 9, to gain a better understanding of the pores, voids, cracks, and micro cracks. For this analysis, ImageJ (version 1.52) software was used, and the scale was the same as the SEM image because the same micrographs were used for this analysis. As shown in in Figure 8a, the control specimen QD0-FNS0 was seen to be comparatively less compact than the other specimens. More pores and voids were visible, which was also justified by the image analysis in Figure 9a, where the blue portion was suggestive of surface voids and cracks. After analysing all the specimens, the surface voids were found to be 12.15, 5.85, 7.25 and 8.30% for QD0-FNS0, QD25-FNS75, QD50-FNS50 and QD75-FNS25, respectively. It can be seen from Figure 8b that the QD25-FNS75 specimen showed the most compact surface in comparison to all the other samples used in this research. This is consistent with the lowest surface void percentage calculated by the software and the highest compressive and tensile strength among all the mixtures. Given that a microstructural specimen with greater compactness is expected to have greater strength [40], this result directly relates with the image analysis for void content. The microstructures in the SEM images displayed varied degrees of compactness, which is consistent with the variations in strength of the mixtures. 

Thus, it is apparent from the image analysis that the control specimen QD0-FNS0 had the highest number of pores and voids, but, on the other hand, the cracks were prominent in the QD75-FNS25 and QD50-FNS50 samples. It is known that higher shrinkage can generate potential cracking in concrete [41]. The shrinkage results from the previous section showed higher shrinkage values in the QD75-FNS25 and QD50-FNS50 samples, which were, thus, consistent with the higher void contents of these samples obtained from image analysis results.

### 3.5. Crystallographic Phase Identification

**Figure 10:**
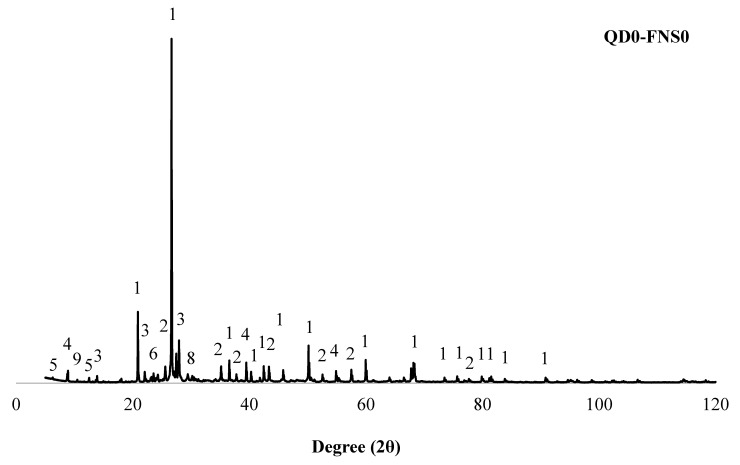

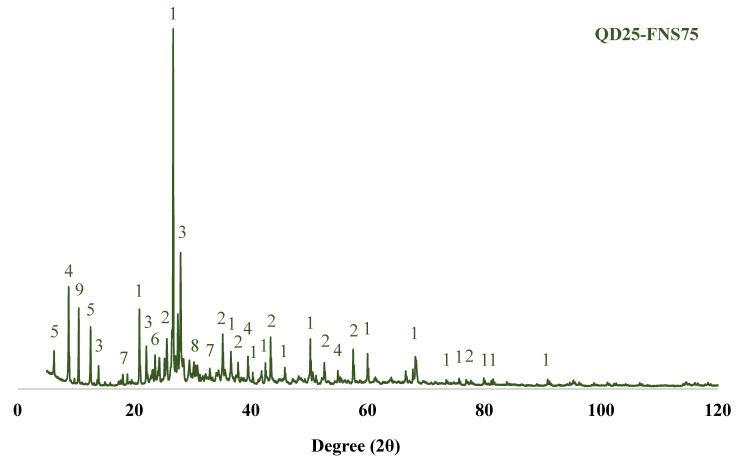

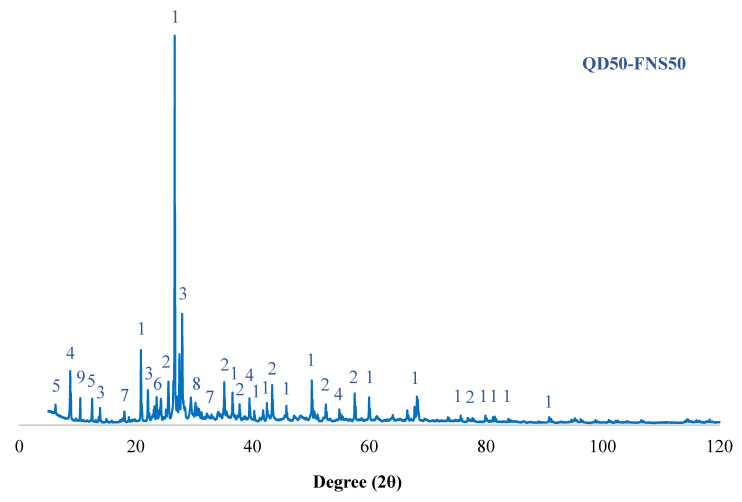

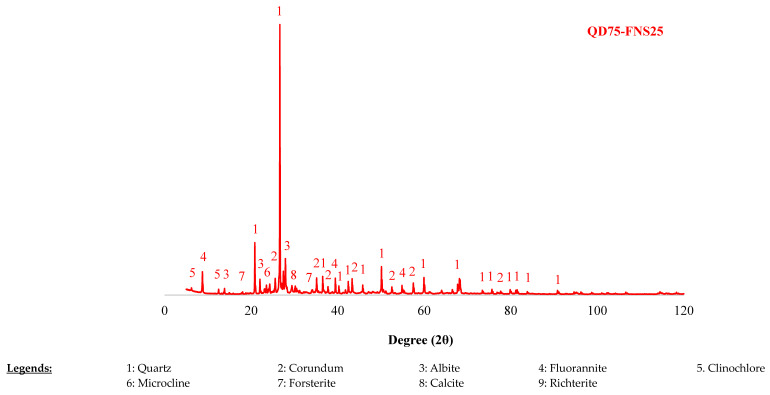
XRD spectra of the studied mixes.

XRD was used to identify the mineral phases in the specimens, and the results are presented in Figure 10. All the specimens contained quartz, which was found from the star matches for the specific peak in the International Centre for Diffraction Data (ICDD) database. Figure 10 shows a sizable amount of corundum, which was added externally as an internal standard material. In addition to these two phases, other phases such as albite, fluorannite, clinochlore, microcline, calcite, and richterite were found in all the mixes. Forsterite was absent in the control mix QD0-FNS0. However, forsterite was present in every other sample, specifically at 17.3 and 35.7 of 2θ. This is explained by the fact that FNS contains an abundance of stable forsterite [17]. Additionally, when the amount of FNS in the mixture decreased, so did the peak intensity for forsterite spectra. The chemical compositions of ferronickel slag from the authors’ previous study [18] make it clear that as the amount of FNS in the mixture decreases, so does the amount of Mg or forsterite in the concrete. No Brucite was found in the reaction product, indicating that the Mg in FNS did not hydrate to create Mg(OH)_2_ but instead remained stable in the form of forsterite. However, from the XRD spectrum, it is evident that the newly formed crystallographic phase albite was higher in QD25-FNS75. The higher strength of the QD25-FNS75 sample can be ascribed to this newly formed phase as albite contributes to gain strength [42]. Fluorannite, clinochlore and richterite phases were also higher in QD25-FNS75. Thus, the XRD findings explain the mechanical strength results well.

## 4. Conclusions

The aim of this research was to investigate the properties of concrete using a combination of nickel slag and quarry dust as the whole substitute of natural sand. The following conclusions present the findings of the study:

- Combining quarry dust and nickel slag provides a well-graded distribution of particles that are compliant with AS 2758.1. This results in a denser packing of particles with less voids in comparison to natural sand.
- Use of quarry dust and nickel slag as fine aggregate produced a higher uniaxial compressive strength of concrete than that using sand. Specifically, QD25-FNS75 had the highest compressive strength, which was 16% greater than the control specimen QD0-FNS0. This is attributed to the rough texture and angular shape of both quarry dust and nickel slag providing a better packing and mechanical interlocking of aggregate.
- Similarly, the combined use of quarry dust and nickel slag produced higher tensile strengths than the control specimen. The mixture QD25-FNS75 produced the highest tensile strength, which was 20% more than the control specimen.
- Mixture QD25-FNS75 had the least shrinkage over the 56 days. The high water absorption of quarry dust caused the QD75-FNS25 and QD50-FNS50 specimens to shrink more than the control specimen.
- In the microstructural investigations, specimens with QD and FNS exhibited fewer voids and a more compact surface compared to the control specimen. This finding is aligned with the mechanical properties observed in this study.

The green concrete concept, emerging towards the end of the previous century, endeavors to substitute some or all components of conventional concrete with recycled or waste materials. Here, this study involved replacing 100% of the fine aggregate with a combination of by-product nickel slag and quarry dust. From the data obtained, it is evident that QD25-FNS75, which uses 25% granite-based quarry dust and 75% nickel slag, is the optimum combination to substitute whole natural sand in concrete. Furthermore, it improves the mechanical properties of concrete, thus affirming its life-cycle sustainability. This conclusion provides a foundation for future research into the benefits of using quarry dust and nickel slag in concrete. The results obtained are promising, and it can therefore be said that based on the limited properties tested, QD25-FNS75 is a viable alternative to sand.

## Figures and Tables

**Table 1:** Fineness modulus of aggregates.

Aggregate Type	Fineness Modulus (FM)
20 mm Coarse	6.96
10 mm Coarse	6
Sand	1.96
Quarry Dust	2.42
Nickel Slag	3.42

**Table 2:** Density and water absorption of aggregates.

Property	20 mm	10 mm	Sand	Nickel Slag	Quarry Dust
Water Absorption (%)	0.40	0.40	0.68	0.48	3.00
Specific Gravity	2.73	2.66	2.6	2.82	2.49

**Table 3:** Concrete mix details (kg/m^3^).

Mix ID	OPC	Fine Aggregate			Coarse Aggregate	Water
		Natural Sand	Quarry Dust	Nickel Slag		
QD0-FNS0	400	750	0	0	1115	180
QD25-FNS75	400	0	187.5	562.5	1115	180
QD50-FNS50	400	0	375	375	1115	180
QD75-FNS25	400	0	562.5	187.5	1115	180

**Table 4:** Relationship between tensile and compressive strengths.

Mix ID	28 Days Splitting Tensile Strength (MPa)		Experimental/Calculated Ratio
	Experimental	AS 3600	
QD0-FNS0	3.59	3.96	0.91
QD25-FNS75	4.29	4.29	1.00
QD50-FNS50	4.12	4.18	0.99
QD75-FNS25	3.91	4.06	0.96

## Data Availability

Data will be provided upon request.
